# Impact of Planning Method (Conventional versus Virtual) on Time to Therapy Initiation and Resection Margins: A Retrospective Analysis of 104 Immediate Jaw Reconstructions

**DOI:** 10.3390/cancers13123013

**Published:** 2021-06-16

**Authors:** Michael Knitschke, Christina Bäcker, Daniel Schmermund, Sebastian Böttger, Philipp Streckbein, Hans-Peter Howaldt, Sameh Attia

**Affiliations:** Department of Oral and Maxillofacial Surgery, Justus-Liebig-University, Klinikstrasse 33, 35392 Giessen, Germany; Christina.Baecker-2@dentist.med.uni-giessen.de (C.B.); Daniel.Schmermund@uniklinikum-giessen.de (D.S.); Sebastian.Boettger@uniklinikum-giessen.de (S.B.); Philipp.Streckbein@uniklinikum-giessen.de (P.S.); HP.Howaldt@uniklinikum-giessen.de (H.-P.H.); Sameh.Attia@dentist.med.uni-giessen.de (S.A.)

**Keywords:** oral cancer, head and neck tumor, fibula free flap, virtual surgical planning

## Abstract

**Simple Summary:**

Computer-aided design and manufacturing of osseous reconstructions are currently widely used in jaw reconstructive surgery, providing an improved surgical outcome and decreased procedural stumbling block. However, data on the influence of planning time on the time-to-surgery initiation and resection margin are missing in the literature. This retrospective, monocentric study compares process times from the first patient contact in hospital, time of in-house or out-of-house biopsy for tumor diagnosis and surgical therapy of tumor resection, and immediate reconstruction of the jaw with free fibula flaps (FFF). Two techniques for reconstruction are used: Virtual surgical planning (VSP) and non-VSP. A total of 104 patients who underwent FFF surgery for immediate jaw reconstruction from 2002 to 2020 are included. The study findings fill the gaps in the literature and obtain clear insights based on the investigated study subjects.

**Abstract:**

Virtual surgical planning (VSP) and patient-specific implants are currently increasing for immediate jaw reconstruction after ablative oncologic surgery. This technique contributes to more accurate and efficient preoperative planning and shorter operation time. The present retrospective, single-center study analyzes the influence of time delay caused by VSP vs. conventional (non-VSP) reconstruction planning on the soft and hard tissue resection margins for necessary oncologic safety. A total number of 104 cases of immediate jaw reconstruction with free fibula flap are included in the present study. The selected method of reconstruction (conventionally, non-VSP: *n* = 63; digitally, VSP: *n* = 41) are analyzed in detail. The study reveals a statistically significant (*p* = 0.008) prolonged time to therapy initiation with a median of 42 days when the VSP method compared with non-VSP (31.0 days) is used. VSP did not significantly affect bony or soft tissue resection margin status. Apart from this observation, no significant differences concerning local tumor recurrence, lymph node, and distant metastases rates are found according to the reconstruction method, and affect soft or bone tissue resection margins. Thus, we conclude that VSP for immediate jaw reconstruction is safe for oncological purposes.

## 1. Introduction

Oral squamous cell cancer (OSCC) is the most common malignancy of the upper aerodigestive tract, with about 90–95% prevalence [1,2]. Five-year survival rates of the progressed staged disease were estimated at 50–60% [3], and its pathohistological residual status (R-category) has been described in the literature as a prognostic factor for tumor recurrence [4,5,6,7,8]. Surgical therapy is aimed to excise the neoplasia with a surrounding safety margin of ≥5 mm (R0-resection) [9], corresponding to an intraoral distance of 10 mm to the palpable tumor border [10]. Further fixation and processing for pathohistological assessment distort measurements, due to tissue shrinkage and, therefore, changes concerning margin evaluation [11].

The margin of excision was defined as close when the distance to the tumor border was between 1–5 mm [12,13]. The involved margin (R1-resection) was described when the distance from the tumor border to the margin of excision was less than 1 mm. This corresponds to an invasive tumor. Close and involved margins were reasons for adjuvant therapy. Published literature concerning resection margins after tumor resection of the oral cavity showed that about 3% were categorized as R1 and 16.3% as close margins [14]. Other investigators found clear margins in half of their collective 896 patients [15]. To increase the safety margin, segmental resection or resection of continuity of the mandible must be performed depending on the location of the tumor site [16,17]. The best time for reconstruction has been debated for some time. Today, immediate reconstruction with osteo-fascio-cutaneous transplants, such as the free fibula flap (FFF) [18], has become the gold standard in maxillofacial reconstruction, due to its advantages and versatility [18,19]. Currently, FFF is the standard therapy for head and neck reconstructive surgery, providing the optimal precondition for later placement of dental implants and, therefore, for oral and dental rehabilitation [20,21]. The immediate insertion of dental implants is sometimes performed simultaneously to jaw reconstruction [22,23,24,25,26]. Nowadays, virtual planned surgery combined with custom-made osteosynthesis—or so-called patient-specific implants, manufactured in a titan laser melting process—has become routine in daily practice. Often pronounced advantages of maxillofacial reconstruction are increased accuracy, decreased operation and ischemia time, reduced length of hospitalization time, improved patient outcomes, highly effective results, and minimized intersegmental gap size [27,28,29,30,31,32,33,34]. Reported disadvantages of VSP are planning time [35], preparation time, and cost aspects, which must be considered [36] as patient-specific (laser-melted titanium) implants are expensive and will decrease the gain on total proceeds.

While high-volume reconstructive centers often use rapid in-house prototyping to manufacture cutting guides and prebending fixation devices. In contrast, low-volume institutions use purpose-made, web-based interfaces for virtual surgical planning of resections and the design of custom-made implants. The time interval of out-of-house service until delivery causes a delay of several days, while tumor growing continues [37,38,39]. Time to therapy initiation (TTI) was a significant influencing factor of upstaging in patients with head and neck SCC (HNSCC) [40].

Published data concerning TTI and quality of excision margins were weak and showed contrary results [27,41,42]. This study aims to estimate the TTI and rate of clean (R0), and close/involved (R1) margins in soft and hard tissue tumor excision when using VSP and PSI for immediate jaw reconstruction with FFF. The study also examines the following oncologic aspects:

Are there differences in the TTI when using a VSP vs. non-VSP (conventional) technique?

What is the influence of resection margins concerning local tumor recurrence, lymph node metastasis, and distant metastasis with respect to the used reconstruction technique?

## 2. Material and Methods

### 2.1. Study Design and Patient Population

The study was conducted as a monocentric, retrospective study. Medical records of patients suffering from SCC who underwent FFF in the head and neck region from January 2002 to December 2020 were analyzed.

Patients were treated with tumor ablation and neck dissection. Immediate reconstruction of the mandibular defect was performed with microsurgical FFF. From 2002 to 2015, reconstructions were conventionally planned (non-VSP) by free-hand during surgery with hand-bent osteosynthesis. Since early 2015, virtual surgical planning (VSP) combined with patient-specific, custom-made implants (PSI) has been performed based on the patient’s preoperative CT scans of the fibula, donor, and tumor recipient site.

Patients were treated according to German guidelines published by Wolf et al. [10].

### 2.2. Study Parameters and Evaluator Calibration

**Figure 1:**
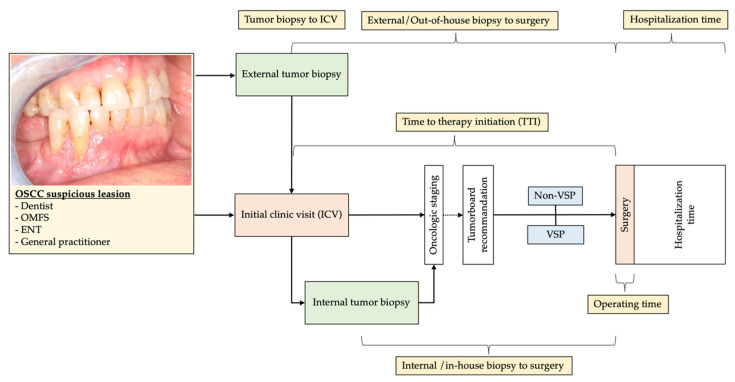
The typical workflow of the first diagnosis of OSCC and referral to oncologic reconstructive OMFS-department and initial clinic visit (ICV). Time intervals of study interest are marked in yellow boxes and correlated to the method of immediate jaw reconstruction.

The following parameters were collected: Age at flap transfer, gender, tumor location, time lapse between diagnosis and surgery, initial clinic visit, time in-house vs. out-of-house biopsy, time to therapy initiation, disease-free survival, pathohistological tumor parameters (T-, N-, and R-Category), planning mode, type of defect [43,44], number of used fibular bone segments, overall length of the fibular flap, and flap outcome (Figure 1). Pathohistological records were assessed and categorized into clear, and close, or involved margins concerning soft tissue and bone. Tumor excision was defined as R0-resection (clear margin) when a surrounding safety margin of ≥5 mm was achieved. Margins were described as close when the distance to the tumor border was between 1–5 mm and involved when it was less than 1 mm (R1-resection).

### 2.3. Inclusion and Exclusion Criteria for Study Subjects

We enrolled patients diagnosed with SCC of the oral cavity for an initial surgical treatment with immediate reconstruction of the jaws with FFF. Only cases with complete data sets and/or medical records were included. Patients with previous chemotherapy, other malignant oncologic diseases, or earlier neoadjuvant irradiation therapy of the head and neck were excluded.

### 2.4. Statistical Analyses

Pearson’s *χ*^2^ test, Fischer’s exact test, and the Freeman–Halton extension [45] were conducted on the categorical variables used to compare both methods of reconstruction concerning gender, external vs. internal biopsy, lymph node involvement, bone invasion and erosion, hard and soft tissue margin, oncologic therapy, number of fibular bone segments, microanastomosis revision rate, and flap success. Student’s *t*-test was performed to compare the mean age at FFF-transfer, tumor diameter, overall length of transplanted FFF, UICC tumor stage, time intervals—tumor biopsy to initial clinic visit and surgery, operating time, and hospitalization time between the study groups after verification of normality. *p* < 0.05 was defined as statistically significant. The statistical analysis was carried out using SPSS 25 (SPSS Inc., Chicago, IL, USA).

### 2.5. Ethics Statement/Confirmation of Patients’ Permission

The local Ethics Committee of Justus-Liebig University Giessen approved the study (AZ27/21), and patients’ permission/consent was not necessary for this retrospective study. The patients consented to their intraoral pictures being used anonymously in the publication. All data in the Microsoft Excel spreadsheet were pseudonymized.

## 3. Results

The tumor database contains 612 records from January 2002 to December 2020. A total number of 104 cases (34 females (32.7%), 70 males (67.3%), mean age of 60.3 ± 9.6 years, age range of 37.0–82.8 years) fulfilled the chosen inclusion criteria for analyses. Secondary reconstruction after tumor recurrence or delayed jaw reconstruction with FFF, as well as cases in which flaps other than FFF had been used, were excluded from this investigation. Over the study period of 18 years, a core team of four, senior, experienced oral and maxillofacial oncologic and reconstructive surgeons performed immediate jaw reconstructions. One surgeon overlooked the whole observation time, the second 16 years, and the other two observed eight years. Collected data were categorized concerning FFF planning procedure: Non-VSP, conventional (*n* = 63) vs. VSP, and digital workflow (*n* = 41). The groups were tested for normal distribution. The detailed demographic parameters and results are summarized in Table 1.

Two-thirds of the whole study group were male. OSCC was mainly localized in the conventional group at the gingiva of an alveolar crest (34.8%) and mouth floor (33.3%). In contrast, in the virtual planned group, the tumor mainly affected the retromolar region (43.9%) and the mouth floor (29.3%). In both groups, about 80% were stratified as UICC tumor stage III or IV. Tumor diameter was measured, resulting in a mean of 37.0 ± 16.4 mm, and all near 4 mm larger than the maximal extension in the non-VSP group. The finding was without statistical significance. Lymph node metastasis was found in both groups in half of the cases. Extracapsular spreading was recorded since 2015 and, therefore, only observed in 14.6% of the VSP-group.

The bone invasion was more frequent in the VSP group (56.1% vs. 50.8%), while bone erosion was slightly more often recorded in the non-VSP group (19.0% vs. 14.6%). Clear bone resection margins (R0-resection) were found slightly more often in the non-VSP group (96.8% vs. 95.1%). The assessment of soft tissue margins revealed clear margins of excision (R0-resection) in 63.4% of patients after virtual and 73.0% after conventional planning. Differences concerning resection margins of the bone (*p* = 0.516) and soft tissue (*p* = 0.470) were not statistically significant.

Surgery only was 52.4%, and surgery in combination with RCT was 28.6% as the treatment of choice in the non-VSP group. In comparison, in the VSP group, surgery only and in combination with adjuvant RT and RCT was performed in a third of the cases.

Defect and FFF-associated parameter data are summarized in Table 2. Maxillary reconstructions have been performed in both groups with a frequency of about 10.0% [44]. The majority of immediate reconstructions were mandible defects. Findings showed a trend in reconstructions of Brown class I and II defects (59.0% vs. 46.4%) with the conventional non-VSP method to class III and IV digitally planned, larger reconstructions (41.4% vs. 33.4%) [43]. The overall length of transplanted FFF was significantly longer in the VSP-group (*p* = 0.002) with a mean of 9.02 ± 2.8 cm. Further, in the VSP-group, polysegmental reconstructions (75.6%) were used significantly more frequently (*p* = 0.014) than in the non-VSP group (52.4%). 

Diagnosis, surgery-associated parameters, and comparisons of intraoperative factor parameter data are shown in Table 3. The time interval from the initial clinic visit (ICV) to surgery was significantly (*p* = 0.008) longer in the VSP group (47.2 ± 24.5 days) compared with the conventional group (35.7 ± 18.6 days).

In both groups, near to two-thirds of the tumor biopsies and initial pathohistological assessments were performed in-house. The time of ICV to surgery showed significant differences when tumor biopsy was performed in-house (*p* = 0.006). VSP was associated with a longer (53.5 ± 26.8 days) lead time compared with conventional planning (36.8 ± 21.1 days). It was insignificant when the tumor was confirmed by external biopsy (*p* = 0.696). The results of ICV to tumor confirmed biopsy showed insignificant differences when the biopsy was conducted externally, with a median of −7.0 days before the patient’s first consultation in the outpatient clinic. In-house tumor diagnosis was confirmed by pathohistological assessment after 5.6 ± 9.0 days in the VSP and after 2.3 ± 4.0 days in the conventional (non-VSP) group; the difference was significant (*p* = 0.045). Time from in-house biopsy to surgical therapy initiation was significantly (*p* = 0.022) longer in the VSP group (47.7 ± 25.2 days) compared with the conventional group (34.5 ± 20.6 days). No statistically significant differences (44.7 ± 15.4 days vs. 44.7 ± 14.1 days) were observed concerning time from out-of-house biopsy to surgical therapy initiation (*p* = 0.995) when both methods of reconstruction were compared.

Operating time resulted in a mean of 508.2 ± 88.8 minutes in the VSP group and was significantly shorter (*p* = 0.005) than in the non-VSP group at 562.5 ± 98.5 minutes. Furthermore, the time from ICV to CT scans of the donor and recipient site were performed slightly earlier in the VSP (recipient site, 10.1 ± 9.5 days; donor site, 14.6 ± 11.6 days) compared with the conventional group (recipient site, 13.4 ± 12.8 days; donor site, 15.7 ± 11.8 days). These observed differences were insignificant. For VSP, the entire turnaround time from kickoff (ICV-kickoff: mean, 21.3 ± 15.6 days; median, 8.0 days) until the delivery of the patient-specific implant (ICV-shipping VSP/PSI: mean, 38.6 ± 18.5 days; median, 35.5 days) was a median of 15.0 days (mean, 16.9 ± 8.5 days).

Hospitalization was found in the VSP group with a mean of 22.5 ± 12.0 days, two days longer than in the non-VSP group. In both groups, the microanastomosis revision rate was lower than 10%. Microanastomosis revision became necessary for flap salvage slightly more frequently in the VSP-group (9.8% vs. 7.9%). Total flap success rate was estimated with 85.4% in the VSP and 90.6% in the conventional group. Differences concerning hospitalization time, microanastomosis revision rate, and flap success were without statistical significance between the groups.

**Figure 2:**
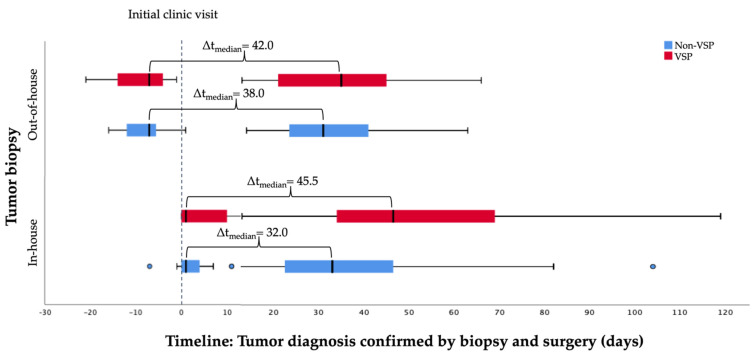
Timeline: In-house vs. out-of-house tumor biopsy to surgery categorized by method of reconstruction planning.

In summary, the shortest time interval between tumor biopsy and surgery at a median of 32.0 days was found when in-house biopsy and non-VSP for immediate jaw reconstruction were performed. If VSP was used, the gap was 45.5 days. Out-of-house biopsy to surgery was estimated with a median time of 42 days in the VSP and 38.0 days in the non-VSP group (Figure 2).

**Figure 3:**
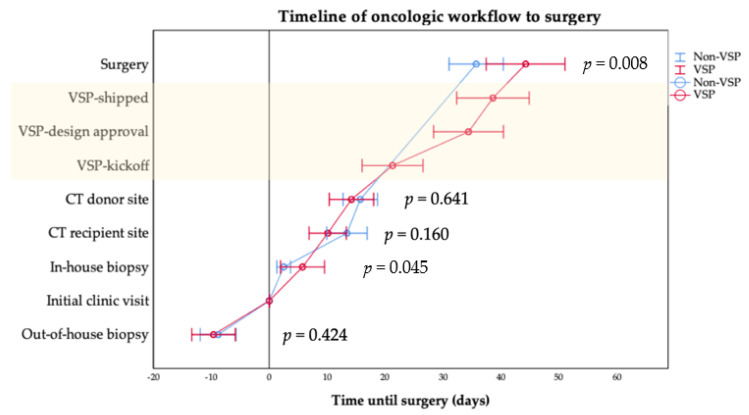
Mean time of diagnostic and planning procedure from the initial clinic visit to surgery categorized by method of reconstruction planning. The 95% confidence intervals are given by whisker bars. Statistically significant differences were found for intervals of the initial clinic visit to in-house biopsy (*p* = 0.045) and surgery (*p* = 0.008) (Table 3). Annotation: The light yellow background marks VSP parameters.

Further, stages of diagnostic and planning procedures from the first contact in the outpatient clinic to surgery were drawn in Figure 3. Overall, the initial oncologic staging procedure was completed after CT scan of the lower limb for transplant planning in both groups near a mean of 15 days after ICV (Table 3). Finally, surgery was performed later in the non-VSP than in the VSP group (non-VSP, 35.7 ± 18.6 days; VSP, 47.2 ± 24.5 days). When VSP was performed, data shows a wide range from VSP-kickoff after ICV was initiated (mean, 21.3 ± 15.6 days; median, 8.0 days). The whole VSP-procedure turnaround time from kickoff to patient-specific implant shipping to our department took an average of 16.9 ± 8.5 days (median, 15.0 days).

**Figure 4:**
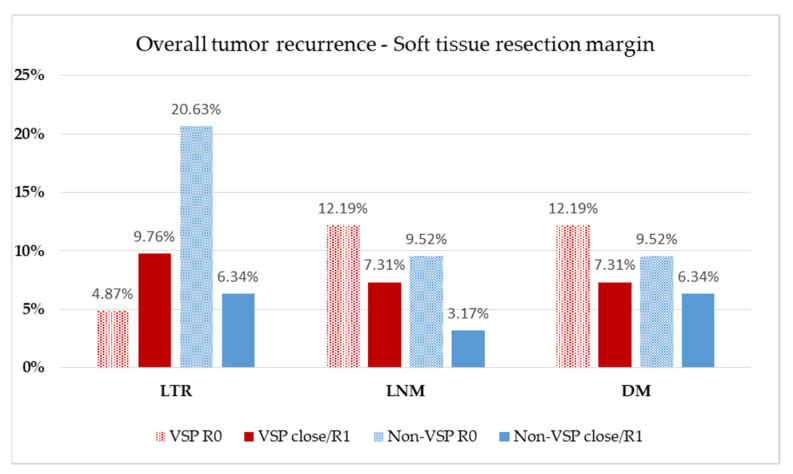
Evaluation of soft tissue resection margin. Relative distribution of local tumor recurrence (LTR), lymph node metastasis (LNM), and distant metastasis (DM) according to the method of reconstruction planning. Patients at risk: VSP group, *n* = 41 (follow-up, 20.6 ± 16.4 months); non-VSP group: *n* = 63 (follow-up, 64.9 ± 52.1 months).

**Figure 5:**
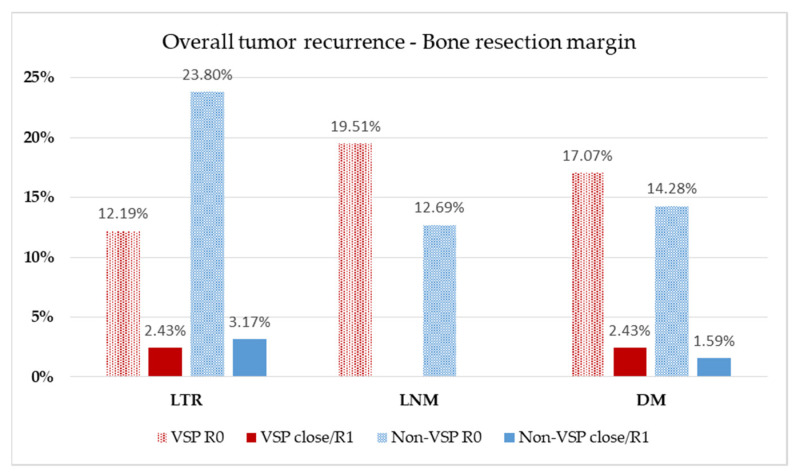
Evaluation of bony resection margin. Relative distribution of local tumor recurrence (LTR), lymph node metastasis (LNM), and distant metastasis (DM) according to the method of reconstruction planning. Patients at risk: VSP group, *n* = 41 (follow-up, 20.5 ± 16.4 months); non-VSP group, *n* = 63 (follow-up, 64.9 ± 52.1 months).

Although differences between the planning groups and resection margins of soft and bony tissue were insignificant (Table 1), 36.6% close and involved margins in the VSP group compared with 26.9% in the conventional, non-VSP group were found. Therefore, the rate of local tumor recurrence (LTR), lymph node metastasis (LNM), and distant metastasis (DM) were evaluated concerning resection margins of soft (Figure 4) and bony tissue (Figure 5) concerning the used planning mode of reconstruction.

For comparison of LTR, LNM, and DM, patients at risk were classified according to the method of reconstruction and assessed on soft tissue margin status (Figure 4) in the recorded follow-up interval (Table 1). A total 14.63% of VSP reconstructions suffered LTR, which occurred about two-times more often when close or involved margins (VSP: R0 = 4.87%, *n* = 2, close/R1 = 9.76%, *n* = 4) were assessed. In the conventional, non-VSP group, LTR was detected in 26.97% of patients and occurred three times more often in the non-VSP group with a clear resection margin than in the close/R1 group (non-VSP: R0 = 20.63%, *n* = 13, close/R1 = 6.34%, *n* = 4). LNM showed 19.51% occurrence in the VSP group and 12.69% in the non-VSP group. In both groups, LNM was slightly more frequently observed in the clean margin group (VSP: R0 = 12.19%, *n* = 5, close/R1 = 7.31%, *n* = 3; non-VSP: R0 = 9.52%, *n* = 6, close/R1 = 3.17%, *n* = 2). DM were found in 19.51% of the VSP group and 15.86% of the non-VSP group. In both groups, DM was slightly more frequently observed in the clean margin group (VSP: R0 = 12.19%, *n* = 5, close/R1 = 7.31%, *n* = 3; non-VSP: R0 = 9.52%, *n* = 6, close/R1 = 6.34%, *n* = 4).

Analogous to soft tissue resection margin, rates of LTR, LNM, and DM, were evaluated for bony resection margin status and also according to the method of reconstruction (Figure 5) about the recorded follow-up interval (Table 1). Together, 14.63% of VSP reconstructions suffered LTR, which occurred about five times more often when clean margins (VSP: R0 = 12.19%, *n* = 5, close/R1 = 2.43%, *n* = 1) were assessed. In the conventional, non-VSP group, LTR was detected in 26.97% of patients and occurred three times more often in the non-VSP group with a clear resection margin than in the close/R1 group (non-VSP: R0 = 23.80%, *n* = 15, close/R1 = 3.17%, *n* = 2). LNM occurred in 19.51% of the VSP group and 15.86% of the non-VSP group. In both groups, LNM was observed despite clean bony resection margins (VSP: R0 = 19.51%, *n* = 8; non-VSP: R0 = 12.69%, *n* = 8). DM were found in 19.51% of the VSP group and 15.86% of the non-VSP group. In both groups, DM was observed more frequently in the clean margin group (VSP: R0 = 17.07%, *n* = 7, close/R1 = 2.43%, *n* = 1; non-VSP: R0 = 14.28%, *n* = 9, close/R1 = 1.59%, *n* = 1).

## 4. Discussion

The free fibula flap (FFF) has become the gold standard in maxillofacial reconstruction, due to its advantages and versatility [18,19]. Vascular pathologies of the lower limb vessels, such as peripheral arterial occlusive disease, stenoses, and vascular anomalies were considered as contraindications for FFF [46]. Preoperatively computed tomography angiography (CTA) [47,48,49,50] or magnetic resonance angiography (MRA) was performed in all cases to ensure three-vessel status to prevent postoperatively critical ischemia of the donor site leg [51,52]. In those cases, patients were excluded for FFF, and alternative reconstructive procedures, such as vascularized deep circumflex iliac artery flap (DCIA) [53], and a hand-bent or customized, patient-specific reconstruction plate [54] was used.

The present study found a significantly longer time to therapy initiation (TTI) when virtual surgical planning (VSP) was used for immediate jaw reconstruction. Concerning TTI, some authors emphasized the necessity for tumor resection during a few days within three weeks after diagnosis and doubted whether this can be achieved with a digital workflow [27]. Compared with the non-VSP group, a significant time delay to TTI was about 12 days in the VSP-group, with a mean therapy initiation at 47.2 days. The observed delay mainly arises from the VSP turnaround time of a median of 15.0 days (mean, 16.9 ± 8.5 days) and time from PSI delivery to surgery of 6.7 ± 4.5 days (median, 5.0 days). However, another study found in their individual setting an insignificant shorter TTI of 59 ± 16 days when VSP was used compared with conventional immediate reconstruction [41]. Published data from the national cancer database of the United States showed that prolonged TTI is currently affecting survival and a TTI of greater than 46 to 52 days introduced an increased risk of death. The risk of death was adverse beyond 60 days [55]. Furthermore, in advanced HNSCC, multidisciplinary treatment is necessary. It was also found that increasing delays in postoperative and radiation intervals were associated independently with an escalating risk of mortality. When the entire treatment period is considered, delays in initiating therapy fade into the background [56]. However, published literature has shown that increased TTI did not impact overall survival [57].

Surgical treatment of oral SCC is based on tumor resection with a circular safety margin, including the continuity of the mandible or extended parts of the maxilla, if tumor invasion was assumed [58]. Immediate microvascular reconstructions have become popular, safe, and reliable [59]. They admit the quality of life, which is comparable to segmental resections of the jaw [60]. While conventional immediate reconstructions have been performed in the past, VSP has become popular in many reconstructive centers. Some high-volume reconstructive centers often maintain their own “in-house” planning and rapid prototype printing devices for bending models or use an “out-of-house” service with the option of a patient-specific implant [37,38,39]. Custom-made (CAD/CAM) osteosynthesis plates are the transfer key that enable three-dimensional configuration of the original and transplanted bone segments [61,62]. The accuracy of the technique has been widely investigated [35,63,64,65]. Our data concur with the results of other studies that the duration of surgery is significantly shortened [35,36,66], due to more accurate and efficient intraoperative fibular osteotomies and faster shaping of the neomandible, and elimination of free-hand contouring and stepwise plate bending [67,68,69]. Statistically significant differences concerning the duration of hospitalization, microanastomosis revision rate, and total flap failures were not found between the observed groups.

However, the crux of VSP is that it is addressed to the snapshot of the CT scan of the recipient site, which is timely localized at the beginning of the staging procedure. The time interval between the CT scan of the tumor (recipient) site and defining the virtual resection planes should be as short as possible to surgery [70]. Prolonged time to therapy initiation (TTI) is a disadvantage of VSP and leads to further tumor growth [37,39,66,71]. Thus, tumor progress should be kept minimal, and involvement of the resection border should be prevented to exclude unexpected modifications of the virtual surgical plan. Possible changes in the resection margins have the consequence that in a two-team approach setting, the shaping of the neomandible must be stopped until the affected bone section has been evaluated. For intraoperative assessment of bony resection margins, flat-panel volume computed tomography could be used to increase tumor-free bony margins [72]. An ever-present risk is that a prepared PSI must be discarded if the bony resection margins change or unforeseen limitations occur on the soft tissue site.

Study findings revealed that CT scans of the recipient (tumor) site were about 10 days older in the VSP group than in the non-VSP group at the time of surgery (VSP, 34.8 ± 17.6 days vs. non-VSP, 25.09 ± 17.2 days). This result is the consequence of the VSP preparation time. In virtual planning, soft and hard tissue tumor involvement is estimated, and a necessary safety distance is mentally drawn. However, the time difference between the CT scan and later virtual planned resection did not have a relevant impact on the frequency of LTR, due to close or involved resection margins during the ongoing follow-up period.

Involved or close resection margins after ablative oncologic treatment increase the risk for local tumor recurrence and development of distant metastasis [73]. Infiltration of the bone correlates with a worse prognosis [74]. An oncologic safety margin of about 5 mm should be the aim for pathohistological classification of free margins status (R0) [9]. Therefore, excision was recommended at about a 10 mm distance around the palpable tumor [10] and 10 mm length to the visible tumor border in the bone. There are some reports in the literature that pathohistological safety distance can be achieved when using the VSP procedure [27,41,42,68,71,75]. Literature shows that rates of local recurrence following margin-free surgical resections can range from 16% to 20% [76]. The failure to obtain clear (R0) surgical margins increases the probability of local tumor recurrence [77]. R1 or R2 are found to occur 1.7 times more frequently in OSCC than in other head and neck subsites [78,79,80]. The study results show that LTR rates concerning clean, soft tissue margins are 20.63% in the conventional non-VSP group, which are well comparable with literature, and are 4.87% in the VSP group. The authors believe that the low rate of LTR in the VSP group was a result of the necessary intensive preoperative oncologic case study for ideal addressing of the tumor location and consequent ablative concept. In our eyes, this is a decisive advantage of the virtual method, aside from the accessible precision of the reconstructions.

To the best of our knowledge, only one study has been published investigating the influence of the time delay on VSP of custom-made implants (PSI) for oncologic reasons and impacts on bony and soft tissue margins [41]. They evaluated *n* = 28 conventional and *n* = 25 VSP primary, immediate mandible reconstructions and found that VSP did not significantly affect bony or soft tissue margin status. Our findings confirm their results concerning the impact of VSP on excision margins. The aforementioned workgroup found, in their individual setting in Ireland, a shorter time interval from clinic visit to surgery in the VSP than in the conventional group (59 ± 16 days vs. 65 ± 30 days) [41], which might be a benefit for the patient. The results of the present study found that the conventional planning method led to surgery faster (35.7 ± 18.6 days) than the virtual method (47.2 ± 24.5 days). The environment of our evaluated university setting revealed a faster time to surgery of around 12 days compared with their TTI. However, the time delay is the critical aspect of the VSP method because tumor progression is time-sensitive [40]. In addition, no differences concerning local tumor recurrence, lymph node, and distant metastases rates were found according to the method of reconstruction that affected soft or bone tissue resection margins.

Furthermore, the time of biopsy must be considered. In both study groups, about a third of the tumor biopsies were performed out-of-house. Process time from biopsy to surgery was almost equal in both groups and independent from the planning method, with a median of 46 days. In contrast to this, TTI was faster when an in-house biopsy for diagnosis was performed. This time advantage supports the recommendation for in-house biopsy at the oncologic and reconstructive center of choice. A median TTI of 43 days was found in the VSP group and 34.5 days in the non-VSP group. However, several influencing variables may bias the extended TTI in VSP. First, the institutional tumor board conference has taken place once a week since 2015. In the specific setting of the department, a schedulable intensive care bed capacity was more or less limited to one day a week. The whole process and production time is a median of 15 days after VSP kickoff and an initial web-meeting. In summary, the longer TTI in the VSP group was mainly influenced by VSP workflow, weekly oncologic board schedule, and limited ICU capacities.

### Limitations of the Study

The retrospective analysis is at risk of various biases. Historical and documentation bias must be considered. Data was extracted out of medical records and the anesthetists’ database. Therefore, transmission error could not be excluded. Pathologists’ reports and assessments were not standardized and had not been prepared for the present investigation. The main focus was on the bone resection margins, but an essential limitation of the current study is the small number of cases involving bony resection margins. However, the findings showed no increase of involved or close resection margins of the bone in our out-of-house setting when virtual surgical planning combined with a patient-specific implant (custom-made osteosynthesis plate) was used. Regarding soft tissue resection margins, a statistically insignificant low increase in close and involved margins was found.

## 5. Conclusions

The present retrospective study is a single-center analysis of 104 cases of immediate jaw reconstruction with free fibula flap, which were conventionally (non-VSP, *n* = 63) or digitally (VSP, *n* = 41) planned. The study revealed a statistically significant prolonged time to therapy initiation in the median of 46.5 days when the VSP method and in-house tumor biopsy were used compared with non-VSP (33.0 days). External biopsies accelerated time from initial clinic visit to surgery (VSP 35.0 vs. non-VSP 31.0 days), but prolonged overall process time from biopsy to surgery. The observed time delay did not significantly affect the soft and bony resection margins. Thus, oncologically-necessary extended resections can be adequately reconstructed using the VSP method. With increasing tumor category, the complexity also increases, leading to a decrease in R0 resections. Nevertheless, we found a lower rate of local tumor recurrence (LTR) under VSP with almost the same rate of lymph node metastasis (LNM) and distant metastases (DM). VSP allows precise immediate reconstruction after ablative oncologic surgery and reduces the entire operation time compared with traditional methods. Further randomized clinical trials are necessary to validate the study findings.

## Figures and Tables

**Table 1:** Demographic parameters of study groups (VSP, virtual surgical planning; FFF, free fibula flap; SD, standard deviation; S; surgery; RT, radiation therapy; RCT, radiation, and chemotherapy).

*N* = 104	VSP FFF (%) 41 (39.4%)	Non-VSP FFF (%) 63 (60.6%)	*p*
Age (years), mean ± SD	62.0 ± 10.0	59.2 ± 9.2	0.146
Follow-up (months), mean ± SD	20.5 ± 16.4	64.9 ± 52.1	0.502
Sex, *n* (%)			
Male	26 (63.4%)	44 (68.5%)	
Female	15 (36.6%)	19 (31.5%)	0.318
Tumor site, *n* (%)			
Gingiva of alveolar crest	6 (14.6%)	24 (34.8%)	
Mouth floor	12 (29.3%)	23 (33.3%)	
Retromolar region	18 (43.9%)	10 (14.5%)	
Maxilla	5 (12.2%)	5 (7.2%)	
Planum buccale	-	1 (1.4%)	
UICC tumor stage, *n* (%)			
Stage I	5 (12.2%)	5 (7.2%)	
Stage II	1 (2.4%)	10 (14.5%)	
Stage III	6 (14.6%)	6 (8.7%)	
Stage IV	29 (70.7%)	42 (60.9%)	
Tumor diameter (mm), mean ± SD	37.0 ± 16.4	33.5 ± 16.0	0.283
Node-positive, *n* (%)	20 (48.8%)	31 (49.2%)	0.562
Extracapsular spread, *n* (%)	6 (14.6%)	-	
Bone invasion, *n* (%)	23 (56.1%)	32 (50.8%)	0.372
Bone erosion, *n* (%)	6 (14.6%)	12 (19.0%)	0.559
Bone margin, *n* (%)			
Clear	39 (95.1%)	61 (96.8%)	
Close	-	-	
Involved	2 (4.9%)	2 (3.2%)	0.516
Soft tissue margin, *n* (%)			
Clear	26 (63.4%)	46 (73.0%)	
Close	10 (24.4%)	13 (20.6%)	
Involved	5 (12.2%)	4 (6.3%)	0.470
Oncologic therapy, *n* (%)			
S	13 (31.7%)	33 (52.4%)	
S + RT	13 (31.7%)	12 (19.0%)	
S + RCT	15 (36.6%)	18 (28.6%)	0.102

**Table 2:** Defect and associated FFF parameters of the study groups (VSP, virtual surgical planning; FFF, free fibula flap; SD, standard deviation) [43,44].

*N* = 104	VSP FFF (%) 41 (39.4%)	Non-VSP FFF (%) 63 (60.6%)	*p*
Maxilla (Brown class), *n* (%)			
II	3 (7.3%)	5 (7.9%)	-
III	1 (2.4%)	-	-
IV	1 (2.4%)	-	-
Mandible (Brown class), *n* (%)			
I	9 (22.0%)	19 (30.2%)	-
II	10 (24.4%)	18 (28.6%)	-
III	14 (34.1%)	20 (31.7%)	-
IV	3 (7.3%)	1 (1.6%)	-
Reconstruction length (cm), mean ± SD	9.02 ± 2.80	7.23 ± 1.83	0.002
Number of segments, *n* (%)			
1	10 (24.4%)	30 (47.6%)	
2	20 (48.8%)	20 (31.7%)	
3	11 (26.8%)	13 (20.6%)	0.014

**Table 3:** Diagnosis, surgery-associated parameters, and comparison of intraoperative factors.

*N* =104	VSP FFF *n* = 41 (39.4%)	Non-VSP FFF *n* = 63 (60.6%)	*p*
Tumor biopsy, *n* (%)			
Internal/In-house	27 (65.9%)	40 (63.5%)	-
External/Out-of-house	14 (34.1%)	23 (36.5%)	0.806
ICV to surgery (days), mean ± SD (median)			
Internal/In-house	53.5 ± 26.8 (46.5)	36.8 ± 21.1 (33.0)	0.006
External/Out-of-house	35.8 ± 15.5 (35.0)	33.9 ± 13.4 (31.0)	0.696
Biopsy to surgery (days), mean ± SD (median)			
Internal/In-house	47.7 ± 25.2 (43.0)	34.5 ± 20.6 (34.5)	0.022
External/Out-of-house	44.7 ± 15.4 (46.0)	44.7 ± 14.1 (46.0)	0.995
ICV to (days), mean ± SD (median)			
Internal/In-house	5.6 ± 9.0 (1.0)	2.3 ± 4.0 (1.0)	0.045
External/Out-of-house *	−9.6 ± 6.2 (−7.0)	−8.0 ± 5.6 (−7.0)	0.424
CT recipient site	10.1 ± 9.5 (8.0)	13.4 ± 12.8 (10.0)	0.160
CTA/MRA donor site	14.6 ± 11.6 (14.0)	15.7 ± 11.8 (13.0)	0.641
Kickoff VSP	21.3 ± 15.6 (8.0)	-	-
Approval VSP	34.4 ± 17.8 (34.5)	-	-
Shipping VSP/PSI	38.6 ± 18.5 (35.5)	-	-
Surgery	47.2 ± 24.5 (42.0)	35.7 ± 18.6 (31.0)	0.008
Oncologic board **	14.9 ± 8.9 (14.0)	-	-
Entire VSP turnaround time	16.9 ± 8.5 (15.0)	-	-
CT recipient site–surgery (days), mean ± SD (median)	34.8 ± 17.6 (32.0)	25.09 ± 17.2 (22.0)	0.008
Operating time (minutes), mean ± SD	508.2 ± 88.8	562.6 ± 98.5	0.005
Hospitalization time (days), mean ± SD	22.5 ± 12.0	20.33 ± 9.0	0.285
Microanastomosis revision, *n* (%)	4 (9.8%)	5 (7.9%)	0.504
Flap success, *n* (%)	35 (85.4%)	57 (90.6%)	0.310

* Annotation: Negative values mean that the biopsy occurred before the initial consultation in the outpatient clinic. ** Interdisciplinary pretherapeutic oncologic board was established in 2015 simultaneously to VSP. (VSP, virtual surgical planning; FFF, free fibula flap; SD, standard deviation; ICV, initial clinic visit; CTA, computed tomography angiography; MRA, magnetic resonance angiography; PSI, patient-specific implant).

## Data Availability

The data presented in this study are available upon request from the corresponding author.
